# Cancer Prevention and Cultural Continuity for Métis Peoples in Canada: A Scoping Review

**DOI:** 10.3390/curroncol31070289

**Published:** 2024-07-05

**Authors:** Maria Diaz Vega, José Diego Marques Santos, Stephanie Witham, Marg Friesen, Tegan Brock, Sheila Laroque, Jennifer Sedgewick, Tracey Carr, Gary Groot

**Affiliations:** 1 Department of Community Health and Epidemiology, College of Medicine, University of Saskatchewan, Saskatoon, SK S7N 5E5, Canadajosediego.marquessantos@usask.ca (J.D.M.S.); tlc143@mail.usask.ca (T.C.); 2 Ministry of Health, Métis Nation—Saskatchewan, Saskatoon, SK S7M 5X8, Canada; 3 Indigenous Studies, University of Saskatchewan, Saskatoon, SK S7N 5E5, Canada; 4 Schulich School of Business, York University Toronto, North York, ON M3J 1P3, Canada

**Keywords:** Métis, cancer, culture, cancer prevention, scoping review, health promotion, Indigenous, Canada

## Abstract

The purpose of this scoping review was to map the literature on the relationship between cultural continuity and health among Métis people as well as how this knowledge could be translated into cancer prevention initiatives. We included any Métis-specific literature evaluating connections between culture, health, and well-being. We conducted electronic searches of Medline, PubMed, Embase, PsychInfo, I-Portal, and hand-searched journals, and reviewed the grey literature. Based on our inclusion criteria, articles were screened and assessed for eligibility, resulting in a sample of 22 publications. Qualitative, quantitative, and mixed methods designs were considered. The 22 publications included in this review were diverse, ranging from population-based studies to reports and news articles. There were no limitations to publication year, and most of the data presented in this review were published more than five years ago. Nevertheless, the results demonstrate the potential role of Métis cultural continuity in cancer prevention. The scoping review revealed the current lack of Métis-specific data regarding health and its intersectionality with culture. However, the existing literature indicates that cultural continuity for Métis appears to influence health and well-being positively. As such, there may be benefits to incorporating cultural continuity in cancer prevention efforts.

## 1. Introduction

Métis are a post-contact Indigenous group in Canada, with a unique society that has distinct cultural, political, and social orientations [1]. As a distinct Indigenous group, the Métis people have unique health needs that require specific attention. Thus, Métis Social Determinants of Health (MSDoH) can be used to comprehend Métis health principles, with the objective of acknowledging the underlying causes of historical, economic, social, ethnic, gender, and political inequities. Some MSDoH include housing, colonialism, education, access to health services, racism, culture, income and social status, employment, and work conditions [2]. Although the Métis have been found to experience poorer health outcomes compared to the overall Canadian population, the effects of past and present colonial policies on Métis health are often underestimated [3]. The impacts of colonization (e.g., the Sixties Scoop, land dispossession due to Métis Scrip) [4] have disrupted cultural continuity for many Métis, affecting their connection with their language, cultural practices, spirituality, family, and generational knowledge passed on by Elders [5,6,7]. The cultural disruption has been associated with several negative outcomes, including heightened risk of depression, suicide, substance abuse (alcohol and/or drugs), poverty, homelessness, and chronic diseases. In addition to leading to poorer health outcomes, these factors have resulted in unequal access to cancer screening and treatment services for Métis people. Furthermore, existing research tends to focus on a pan-Indigenous approach, ignoring the specific context and needs of different Indigenous groups. These contextual factors, including variations in culture, knowledge, and language, can act as barriers to cancer screening participation, potentially increasing cancer rates among Métis people [8].

Connection to culture has been highlighted as an important factor for the health and well-being of Métis people [5,9]. Several studies have emphasized that (re)connecting and strengthening relationships to culture has a positive influence on the health of Métis people [5,9,10]. For example, a community-based participatory study in a Métis Region of Alberta found that connection to culture, land, and ancestry was associated with individual well-being [10]. Similarly, a qualitative study with Métis participants in British Columbia highlighted the importance of cultural continuity on mental health [9]. Together, these studies demonstrate the link between cultural continuity, well-being, and healing for Métis people [5,10,11].

Because culture is closely related to healthy living (or living a good life), there is growing interest in understanding the role of culture in cancer prevention among Métis. Robust evidence indicates that healthy living can prevent and decrease cancer mortality [12]. Healthy behavioral factors (e.g., avoiding smoking and harmful alcohol use, maintaining a healthy weight, exercising, and eating well) can lead to a 29% and 52% reduction in the risk of incident cancer and cancer mortality, respectively [12], suggesting that adopting healthy behaviors is the “best buy” in cancer prevention.

Several factors, such as tobacco use, obesity, and lack of cancer screening, have been found to contribute to higher cancer rates among Métis people [13]. Métis people also experience lower cancer survival rates due to barriers to accessing healthcare. These factors contribute to cancer being a leading cause of death among Métis people.

The growing burden of cancer among Métis people, along with significant knowledge gaps in data about Métis health and cancer prevention efforts [14,15], warrant further exploration into prevention strategies designed by and for Métis people. A recent scoping review of the literature, programs, policies, and educational resources for Métis people with cancer in Canada highlighted the need for Métis-specific cancer prevention strategies [15]. Métis-specific cancer prevention strategies align with the Truth and Reconciliation Calls to Action (TRC) #20 and #22, which pertain to the need to address the distinct health needs of Indigenous Peoples and to call and endorse the value of Indigenous healing practices for health and well-being.

A previous scoping review on Métis people and cancer [15] revealed a lack of Métis-specific information. Therefore, a scoping review was utilized to capture broader evidence, showing a more diverse range of findings related to cancer and health promotion efforts within this group. Additionally, this method enabled us to identify the gaps in the existing Métis-specific literature, highlighting areas where further research is needed [16]. The findings of this study have enabled us to address the following research question: In what ways does cultural continuity lead to health promotion and cancer prevention for Métis people in Canada?

### Scoping Review Objectives

The primary objective of this scoping review is to map the literature on cultural continuity for Métis people and its role in health promotion and cancer prevention in Canada. Specifically, the review will

1. Determine the extent, nature, and range of research activity on cultural continuity for Métis people, health promotion, and cancer prevention.
2. Summarize how cultural continuity leads to health promotion and cancer prevention for Métis people.

## 2. Materials and Methods

The following six stages of the Preferred Reporting Items for Systematic Reviews and Meta-Analyses Extension for Scoping Reviews (PRISMA-ScR) guidelines were followed: (1) identification of the research question; (2) identification of relevant studies; (3) selection of eligible studies; (4) charting the data; (5) collating, summarizing, and reporting of the results; and (6) consultation with stakeholders [17]. This review draws from other established frameworks and guidelines for scoping reviews: Arksey and O’Malley in 2005 [18], Levac and colleagues [19], and the Joanna Briggs Institute (JBI) framework for scoping reviews [16].

**Stage 1: Identification of the Research Questions**

The research question is as follows: In what ways does cultural continuity lead to health promotion and cancer prevention for Métis people in Canada?

**Stage 2: Identification of Relevant Studies**

This review used the Population, Concept, Context (PCC) framework outlined by the JBI [16]. We based our search strategy on the PCC framework described in Table 1.

Search Strategy

The search strategy utilized cancer search terms and aimed to locate primary studies, reviews, and grey literature. We also applied a filter of search terms for Indigenous Peoples in Canada developed by the University of Alberta (2022). The search strategy included keyword index terms and Medical Subject Headings (MeSHs) for PubMed and Medline [20]. The searches occurred between July and September 2022.

The literature search was conducted using the following databases: Medline, PubMed, Embase, PsychInfo, and I-Portal (University of Saskatchewan’s Indigenous Studies Portal). The grey literature search screened the USask I-Portal, provincial and federal government websites, Métis organization websites, mainstream media news articles, Google Scholar and general search engines, clinical health guidelines, and relevant Indigenous organization websites.

**Stage 3: Selection of Eligible Studies**

This review considered English publications that included Métis people. We felt that it was important to privilege Métis culture and avoid a pan-Indigenous approach. Any relevant Métis-specific literature, regardless of the age or gender of participants, was included. This review considered publications about Métis culture, health, and well-being, including language learning, ceremony, cultural practices, knowledge transmission, and other aspects of cultural continuity. This scoping review also considered quantitative, qualitative, and mixed methods study designs, as well as review articles and grey literature (e.g., reports, newspaper news) for inclusion. No limitations were applied to publication dates.

All papers and grey literature documents were uploaded into Mendeley reference management software (https://www.mendeley.com/, accessed on 11 June 2024) [16]. After search completion, a PDF of each publication was uploaded from Mendeley to the Covidence systematic review manager [21,22]. The abstracts and the full-text versions of the literature were assessed using the inclusion/exclusion criteria developed by the research team. Publications were included if they described Métis cultural practices and/or cancer prevention for Métis people and were excluded if they referred to all Indigenous Peoples in Canada or medical treatments. All grey literature, including videos and presentations, was included for review. Any disagreements that arose between reviewers were discussed with a third reviewer.

**Stage 4: Charting the Data**

A data extraction tool was developed and tested by the reviewers. This tool was used by two independent reviewers. Utilizing Covidence, data extraction was performed using a piloted data extraction table to ensure that the collected data and themes were consistent with the research question and purpose. Based on Arksey and O’Malley’s [18] and Levac et al.’s [19] guidelines, the data extraction table included the following elements:

(1) Author(s), year of publication, and study location;
(2) Intervention and comparator (if applicable);
(3) Study populations;
(4) Aims of the study;
(5) Health outcomes;
(6) Methodology;
(7) Outcome measures;
(8) Important results.

**Stage 5: Collating, Summarizing, and Reporting the Results**

The results from the previous stages were synthesized into tables to provide an overview of the literature on Métis cultural continuity and health promotion that contributes to cancer prevention. We also synthesized the evidence in a narrative format to explain how the study results complemented each other. A figure showing the enablers and barriers to cancer prevention among Métis was developed.

**Stage 6: Consultation with Stakeholders**

Once the review protocol was solidified, a research advisory committee was created. This committee comprised representatives from the Métis Nation-Saskatchewan (MN-S) Health Department, Métis citizens (community members), the Saskatchewan Cancer Agency, and the University of Saskatchewan. To represent the Métis community, a Métis knowledge keeper, a Métis cancer survivor, and a Métis youth also joined this committee to inform us about how to best translate the study results. The research findings were summarized and presented to the committee in lay language. Based on the preliminary results of this scoping review, we prepared a lay language annotated bibliography and PowerPoint presentations to share the research findings and possible knowledge translation strategies with the committee. The committee discussed several ideas for knowledge dissemination strategies that could be impactful for Métis communities. As a group, we chose to show the importance of Métis cultural continuity and its impacts on health, well-being, and cancer prevention by developing a video that is owned and controlled by MN-S. The video starts with a short description of the research projects that have been completed in collaboration with MN-S. Next, the video depicts a Métis chef and her mentor harvesting and cooking to demonstrate the importance of a traditional diet. The final section of the video shows Métis cultural dancers doing a performance to showcase Métis jigging to show how cultural activities can promote exercise. Métis jigging combines First Nations dancing and Scottish and French-Canadian step dancing, and reel, jig, and quadrille steps [23]. It originated in the Red River area, which flows north through southern Manitoba and into Lake Winnipeg [24]. As suggested by the committee, we will also prepare a webinar on the importance of culture for cancer prevention to be live-streamed on the MN-S Facebook page in April 2024.

## 3. Results

### 3.1. Study Inclusion

**Figure 1:**
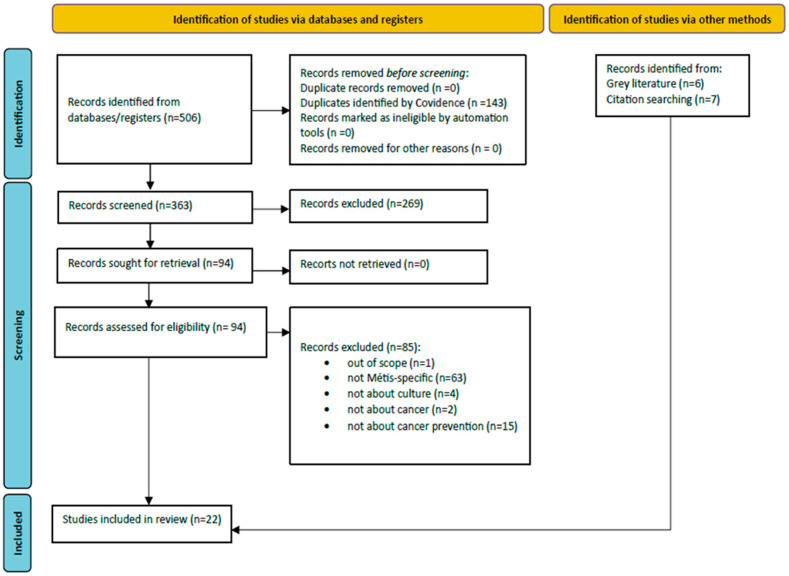
Flowchart of study identification, screening, and inclusion process.

As outlined in Figure 1, the database search resulted in 363 records after eliminating duplicates. Following title and abstract screening, 269 records were excluded. Of the 94 records that remained, 85 were eliminated for not meeting eligibility criteria. Therefore, nine records from the database search were included. An additional seven records were included after citation searching and six records were included from the grey literature. Thus, a total of 22 records were included in the review.

#### Characteristics of the Included Studies

As depicted in Table 2, 10 records were published ≤ in 2014 and 12 were published ≥ in 2015. Most publications were from Canada-wide studies (*n* = 8), followed by publications from Saskatchewan (*n* = 6), Ontario (*n* = 2), Manitoba (*n* = 2), and Alberta (*n* = 2).

### 3.2. Review Findings

This section starts with a brief discussion of the issue of limited Métis-specific health data. We then briefly mention some examples of rates of cancer and screening as well as behavioral factors relevant to cancer prevention, namely, smoking, drinking, physical activity, and obesity, among Métis. Finally, the results about the relationships between Métis culture, health promotion, and cancer prevention are presented.

### 3.3. Lack of Métis-Specific Health Data

The lack of Métis-specific health data is an important finding of this scoping review [21,22,34]. Data pertaining to Métis health and culture are limited, posing a challenge for culturally appropriate cancer prevention strategies [15,26,29,42]. For example, there is minimal information on colorectal cancer among Métis even though Métis females have significantly higher mortality from “intestine and rectum” cancer than their non-Aboriginal counterparts [25].

### 3.4. Cancer Rates

Although Métis-specific cancer data are limited, in one study, Mazereeuw et al. (2018) examined data from the 1991 Canadian Census Health and Environment Cohort. The authors evaluated cancer incidence and survival among Métis adults in Canada and found that Métis adults presented higher rates than the overall population in Canada of the following cancers: gallbladder, liver, cervix, lung, larynx, and female breast [13].

### 3.5. Cancer Screening

In a study by McDonald and Trenholm 2010 et al., Métis presented similar rates for the Papanicolaou test in the last 12 months (49.2 vs. 55.4) and in the last 3 years (80.2 vs. 80.1) compared to those of the general population. However, the rate of mammograms in the last 2 years (39.2 vs. 50.6) was much lower for Métis compared to that of the general population. The age range for mammography screening was 50 to 65 years, and the age range for Papanicolaou tests was 21 to 65 years [26].

### 3.6. Smoking and Drinking

Smoking and drinking appear to be higher among Métis compared to the general population. For example, McDonald and Trenholm (2010) analyzed data from the 2000–2001 and 2004–2005 Canadian Community Health Surveys and the 2001 Aboriginal People’s Survey produced by Statistics Canada. The authors found that Métis reported higher smoking rates (42.7%) compared to the non-Aboriginal population (23.7%) [26]. In McDonald and Trenholm’s (2010) study, Métis reported higher rates of binge drinking in the last month (18.9%) compared to the non-Aboriginal population [14.1%].

### 3.7. Physical Activity and Obesity

A study by Ryan et al. (2018a), it was found that 63.7% of Métis adults were classified as overweight or obese. The authors reported that 51.3% reported leisure time physical activity (doing physical activities outside of work) of less than 3 h/week, and 48.7% reported physical activity of 3 or more hours/week [35]. Time engaging in active transportation (walking) was reported to be 33.6% (less than 1 h/week), 38.5% (1–5 h/week), and 27.9% (more than 5 h/week) [35].

### 3.8. Relationships between Métis Culture, Health Promotion, and Cancer Prevention

Spirituality is a strong component of Métis culture. Kumar and Janz (2010) presented data from the Métis Supplement survey and highlighted that about one-fifth of Métis (21%) consider themselves “very” spiritual or religious, while another 43% consider themselves “moderately” spiritual or religious. On the other hand, approximately 20% of Métis do not consider themselves to be very spiritual or religious, and 10% of Métis do not consider themselves to be spiritual or religious. Métis spiritual and religious practices vary greatly. For instance, some Métis maintain their religious or spiritual health by praying (36%), attending church (30%), meditating (20%), talking with Elders (15%), participating in pilgrimages (5%), attending sweat lodges (4%), or engaging in other activities to maintain their religious/spiritual health [36]. Sweat lodges are heated structures Indigenous peoples use for purification rituals and promoting healthy living. The intense heat promotes sweating out toxins and negative energy to cleanse the body, mind, and soul [43].

In Ryan et al.‘s (2015) study, Métis individuals who reported high levels of spirituality were less likely to smoke [28]. Métis individuals who belonged to a Métis cultural organization smoked less frequently than those who did not [28].

In a focus group utilizing a “talking circle” methodology, Métis women referred to health as more related to physical issues, while “well-being” was defined as broader and included physical, mental/intellectual, emotional, and spiritual aspects [33]. Conceptions of health included sustenance with activities that included exercise, a healthy diet, and an understanding/acceptance of changes in health with age (i.e., functional decline) [33]. Conceptions of well-being included holistic perspectives and collectivity as opposed to individualism, with spirituality through prayer as its most significant practice [33].

Similarly, Ginn et al. (2021) explored Métis health, spirituality, and well-being using a survey and found that connection and balance in health, mental, emotional, spiritual, and physical components were central to well-being [10]. One participant explained this connection, asserting “How are [health, spirituality and well-being] separated—one cannot be without the others—spirituality is the nucleus or focus which keeps all others in balance and sync” [10].

According to Ginn et al. (2021), connection to culture can be categorized into four types: (1) connection to Métis ancestry (Métis awareness and sense of belonging); (2) connection to community (wholeness through connection with culture and community); (3) connection to land (passing on historical knowledge and traditions); and (4) connection to tradition (Indigenous and spiritual practices) [10]. Regarding the connection to Métis ancestry, a participant stated “If I hide my Métis identity, my spirit and my health suffer” [10].

Regarding traditional medicine, in a survey administered by MN-S to Métis citizens, 42.1% of participants knew about traditional healing services in their community, but only 19.2% had accessed them [40]. When asked about activities engaged in to improve health, many participants reported exercising and walking as well as hunting and fishing. Participants also valued fresh air and being outdoors [40]. According to participants, healthy eating was vital and a healthy diet included fresh fruits and vegetables, vitamins, and wild meat [40]. Tobacco, alcohol, or substance use cessation were considered ways to live healthier [40].

Other factors also contribute to good health, such as relationships with family, access to healthcare, spirituality or a positive attitude, education, living in rural areas, traveling, and participating in cultural activities [40]. Participants were also asked about resources in the community that keep them healthy and mentioned access to recreational facilities, including gyms, cultural camps, a pollution-free environment, appropriate housing, and safety. One participant reported on the resources available, explaining that there are “lots of things to do here. Outdoor activities, clean air, water quality, fresh fish. Church/community events. Powwows/Round Dances and Lunch Box Socials. Hockey games. Our community is very supportive and tries to meet everybody’s needs” [40]. Similarly, a Métis participant also attested to the importance of cultural activities for healthy living, stating that “fiddle and jigging as exercise, it brings people together. We need to get people out socializing” [41].

Through community-based workshops, the MN-S found that cultural activities, such as fiddling and jigging, can promote exercise and bring people together [40]. Community members emphasized the need for “food and fun” to improve health. The community also highlighted the need for cultural health promotion activities to raise awareness about disease prevention. For example, a community member highlighted the need for workshops about the harms of “junk food” and reading food labels. Inter-generational learning was also highlighted as a way to pass on knowledge of healthy living from Elders to children [39]. Developing and implementing these initiatives requires strong collaboration with community partners [40].

In an interview, Métis elders stressed the need to highlight Métis culture and pass it on to the next generations [41]. For example, Elder Peter Durocher grew up in Île-à-la-Crosse (predominantly Métis community) and was immersed in Métis culture. As a grandfather, Elder Peter stressed the need to pass on the culture of playing games (e.g., nail-pounding, trap setting, snowshoe races) to the next generations, sharing how “All you can do is your best, and share a little bit of your knowledge with people who don’t know what they’re doing […] These games are something that our ancestors did for so many years, and it’s great for kids to carry on” [41]. Elder George Malboeuf also spoke on the importance of cultural activities [41]. The Elder attended cultural gatherings and races whenever possible, claiming that participating in competitions is a way to keep in good shape [41].

### 3.9. Proposed Framework

**Figure 2:**
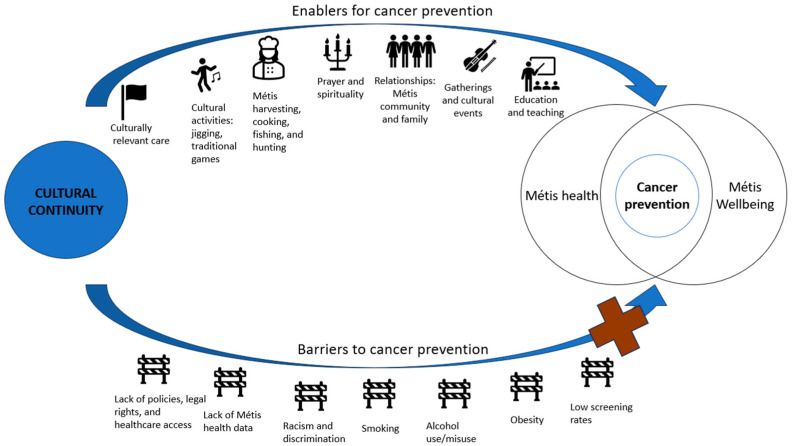
Métis framework for health promotion and cancer prevention through cultural continuity.

A Métis framework for the associations between cultural continuity, Métis health and well-being, and cancer prevention is shown in Figure 2. Although indirect, based on the literature, we argue that cancer prevention is related to Métis health and well-being; thus, the cancer prevention circle is placed where the Métis health and Métis well-being circles intersect [25,37]. To achieve cancer prevention through cultural continuity, we identified several enablers: culturally appropriate care [15,30,37,42]; cultural activities [10,40,41]; Métis harvesting and cooking [37,40]; prayer and spirituality [10,25,36]; relationships [39,40]; gatherings and cultural events [40,41]; and education and teaching [38,40]. On the other hand, several barriers jeopardize cancer prevention through cultural continuity, namely, lack of policies, legal rights, and healthcare access [1]; lack of Métis health data [1,15,34]; racism and discrimination [30]; smoking [26,27,28,32]; alcohol use [26,31,40]; obesity [35]; and low screening rates [26,40]. As a whole, this proposed framework outlines the main factors to consider when applying cultural continuity to prevent cancer for Métis.

## 4. Discussion

This scoping review maps the literature on cultural continuity for Métis people and its role in health promotion and cancer prevention in Canada. Our search for publications on Métis culture, health promotion, and cancer prevention rendered few results relevant to the research question. The 22 publications found in this review are diverse, ranging from population-based studies to reports and news articles. Most of the data presented in this review were published more than five years ago. Nevertheless, consistent with the identification of culture as a key MSDoH [2], the results demonstrate the potential role of Métis cultural continuity in cancer prevention.

One possible way to incorporate Indigenous culture into cancer prevention strategies is to develop culturally appropriate programs. For example, culturally tailored smoking cessation and prevention strategies have been developed for American Indians (AIs) [44,45]. Adult AIs may prefer interventions that are tailored to their culture, as cultural tailoring may help people quit smoking [44]. In a study of 300 never-smoking and ever-smoking urban AI youth in Minneapolis–Saint Paul, it was found that referring to American Indian cultural values may be a useful strategy for the prevention or cessation of smoking [45]. Similarly, exposure to a culturally specific educational message about the sacredness of traditional tobacco can decrease several behavioral factors related to smoking, such as smoking intention [45]. On the other hand, conventional messages about smoking cessation did not seem to alter people’s opinions about smoking [45]. Therefore, interventions that include the cultural consequences of smoking may be more successful than those that are not clearly connected to or are inconsistent with Indigenous cultures [45].

Another modifiable risk factor for cancer is alcohol use, which has been found to be high among Métis people. There is a positive correlation between the duration and quantity of alcohol consumption, even in moderate amounts, and the likelihood of developing cancer, which is associated with the development of oropharyngeal and larynx cancer, esophageal squamous cell cancer, hepatocellular carcinoma, breast cancer, and colorectal cancer [46]. Andersen reviewed alcohol treatment options for Indigenous people and reported that Western medicine may be less effective for Indigenous populations [47]. On the other hand, stories, talking circles, sweat lodges, spiritual practices, and the reconstruction of one’s Indigenous identity and values have all been recommended as traditional alcohol healing practices [47]. This culturally appropriate intervention has the potential to reduce alcohol use among adults. However, Métis youth may be a more desired population group when exploring upstream approaches to cancer prevention.

To better address behavioral factors related to cancer prevention, interventions to prevent cancer should start at an early age. Regular exercise can enhance cardiorespiratory fitness, strengthen bones and muscles, manage weight, reduce anxiety and depression, and reduce risks of several health conditions, such as obesity and cancer [48]. Indigenous cultures, traditions, and connections to the land have the potential to increase access to sports and other activities [49]. McHugh et al. claim that similar to the connection to culture, the physical activity experiences (e.g., traditional games, fishing, and preparation of foods) of Indigenous youth in Canada are fundamentally defined by traditional sport and recreation. In fact, Indigenous youth may perceive physical activities as including chores, recreation, and cultural activities [49]. Likewise, for Métis people, cultural activities that involve exercise and recreation (e.g., traditional games, jigging, hunting) can promote physical exercise. When these cultural activities are practiced at an early age, they have the potential to become a regular practice throughout adulthood, revitalizing culture and promoting health.

Of note, physical activities can provide Indigenous youth with opportunities to develop and maintain relationships with family and community. Developing relationships through physical activity is considered a strengths-based approach that enhances a sense of hope. In this context, hope is developed by finding strengths and building up relationships with people, community, and society [50]. These relationships can increase perception of one’s abilities, confidence, and judgment [50]. In physical activity promotion, identifying strengths associated with physical activity participation and connecting with other individuals, communities, and cultures can foster hope for the improvement of health and well-being.

Using culture to promote physical activity and prevent cancer among Métis is also about promoting Métis cultural continuity and reconciliation. In the past, Aboriginal Peoples lived healthy, active lives, in which hunting, fishing, gathering, and preparing food were part of their daily routine [51]. Traditional sports like lacrosse, wrestling, jogging, kayaking, archery, dancing, storytelling, and singing were all popular [51]. Aboriginal Peoples maintained physical strength, fitness, and health from youth to old age with these practices [51]. Currently, a crucial aspect of the reconciliation process involves the decolonization of sports and recreation. In fact, five of the ninety-four Truth and Reconciliation Calls to Action address sports and reconciliation (calls 87–91) [52,53]. Briefly, these calls pertain to the inclusion and support of Indigenous athletes in Canada’s sport agenda, while also considering culturally appropriate strategies to promote physical activity among Indigenous peoples.

The importance of involvement in cultural events is a key finding about the health of Métis people. Attending cultural events can bring feelings of pride, joy, and reaffirmation of Métis identity [10,40,41]. The holistic advantages of engaging in physical activity encompass a sense of cultural connectedness, as well as the acquisition and transmission of traditional practices [49,52]. Cultural events can provide an opportunity for Métis to connect with each other and celebrate their community and achievements with food and entertainment, contributing to their health and well-being. Attending Métis cultural events not only provides an opportunity to feel healthy and vibrant as a community, but it can also provide a space that prioritizes Métis resurgence [54].

This scoping review demonstrates the importance of spirituality and prayer for the health and well-being of Métis people. For example, in one study a Métis Elder explains the connection between spirituality and health through a Métis lens, referring to spirituality as a balance not only for an individual but also for their family, community, society, and the world and beyond, including the spirit world [55]. Additionally, Métis Elders stated that a beneficial strategy to achieve more balance and improve one’s ability to live a good life is to be situated in a sacred circle of life in relation to Mother Earth, maintain a relationship with the Creator, and express gratitude for this relationship [55]. For instance, fasting is one ceremony that can help a person feel more connected to spiritual activities and more aware of their dual natures as spiritual and physical beings, contributing to healing [55].

Strength-based approaches offer a heightened potential to improve overall health, promote general well-being, and prevent cancer among Métis. In this context, we argue that cultural continuity can be applied using strength-based approaches [50] that privilege Métis culture and emphasize the inherent strengths of Métis individuals, families, and communities. This approach aims to foster self-empowerment and self-determination in relation to health and well-being. By adopting a strengths-based perspective, it is possible to cultivate a sense of optimism by fostering health promotion outcomes associated with [50], for example, engaging in regular exercise through Métis games or jigging.

There are several limitations to this scoping review. First, there is a lack of studies that depict the relationship between Métis culture and cancer prevention. However, we considered publications that had implications on cancer risk factors and their relationship with Métis culture, including health and well-being. Second, we did not assess the quality of the studies included, as per scoping review guidelines. Therefore, the final sample was mixed with different publications and levels of evidence that may or may not complement each other. Nevertheless, this study integrates Métis cultural continuity, health promotion, and cancer prevention, with relevant evidence to inform health promotion strategies. Finally, despite our attempts to develop search strategies that capture all relevant research, some may be missed, as language could also be a limitation for not including French and Michif.

## 5. Conclusions

In conclusion, this scoping review of the relationship between cultural continuity and health among Métis in Canada demonstrates the benefits of incorporating culture into health promotion efforts. Cultural activities that provide opportunities for Métis to build relationships and connect to their Métis identity and tradition could have positive effects on overall health and utility in cancer prevention initiatives. However, this scoping review also reveals the current lack of Métis-specific data regarding health and its intersectionality with culture. Future research should further examine the connection between culture and Métis in both health promotion and cancer prevention.

## Figures and Tables

**Table 1:** Population, Concept, Context (PCC) framework used in the scoping review.

PCC element	Definition	Example
Population	Métis People in Canada	Citizens of Métis Nation Saskatchewan, Citizens of Métis Nation Alberta
Concept	Cultural Continuity	Community events
Context	Health Promotion	How positive health outcomes can lead to cancer prevention

**Table 2:** Details of the studies included in the scoping review.

Author (Year)	Setting	Objectives	Methods	Sample Size
**Academic Sources**				
(Brock et al., 2021) [15]	Canada	To summarize the available data, knowledge, and cancer continuum support for Métis people across Canada	Scoping review	77 records
(Barlett et al., 2011) [25]	Manitoba	To describe the cancer burden on Manitoba’s Métis	Quantitative research using data from the Métis Population Database, the Canadian Community Health Survey, and the National Population Health Survey	73,000 Métis people
(McDonald and Trenholm 2010) [26]	Northern Canada	To examine the disparities in health-related behaviors and use of medical services associated with cancer incidence and diagnosis between Inuit and other people (Métis) of Northern Canada	Quantitative research using data from Statistics Canada	26,585 residents of Northern Canada
(Kewayosh et al., 2015) [27]	Ontario	To report on a strategy to reduce cancer health inequity among Indigenous people in Ontario	N/A	N/A
(Ryan et al., 2015) [28]	Canada	To examine the correlations between current smoking and culturally specific factors among Métis	Quantitative research using data from the 2006 Aboriginal Peoples Survey	6610 Métis adults
(Sanchez-Ramirez et al., 2016) [29]	Alberta	To investigate the incidence and mortality burden of cancer among Métis and to compare disease estimates with non-Métis populations	Quantitative research using data from the Alberta Ministry of Health	3,791,248 Alberta residents
(Sedgewick et al., 2021) [30]	Saskatchewan	To determine the support requirements of Indigenous cancer patients and their families from the perspective of cancer service providers	Focus groups	20 cancer service providers
(Withrow, Amartey, and Marrett 2014) [31]	Ontario	To examine the prevalence of cancer risk factors and screening behaviors among three populations in Ontario, Canada: off-reserve First Nations, Métis, and non-Aboriginal populations	Quantitative research using data from the Canadian Community Health Survey	990 Métis adults
(Mazereeuw et al., 2018) [13]	Canada	To analyze site-specific cancer incidence and survival rates among Métis people in Canada, comparing their risk to non-Aboriginal populations	Quantitative research using data from the Canadian Census, the Canadian Mortality Database, and the Canadian Cancer Registry	2,663,820 Métis Canadians
**External Sources**				
(Cawley et al., 2018) [32]	Ontario	To develop indicators of commercial tobacco exposure as a risk factor for cancer for Indigenous people living in Ontario (on and off-reserve)	Quantitative research using data from the Canadian Community Health Survey and the Ontario First Nations Regional Health Survey	139,336 Indigenous residents of Ontario
(Bartlett 2005) [33]	Manitoba	To understand Métis women’s perceptions of health and well-being	Focus groups/talking circles	17 Métis women living in Manitoba
(Canadian Partnership against Cancer 2009) [34]	Canada	To report on gaps in accessing cancer care for Aboriginal Peoples	Report on a national forum	N/A
(Ryan et al., 2018) [35]	Canada	To investigate the correlates of leisure time physical activity and active transportation among Métis adults	Quantitative research using data from the 2006 Aboriginal Peoples Survey	5810 Métis adults
(Ginn et al., 2021) [10]	Alberta	To explore health, spirituality, and well-being among Métis	Survey	29 Métis residents of Alberta
(Macdougall 2017) [1]	Canada	To report on Métis health and well-being considering land, family, and identity	Report on Métis health	N/A
(Kumar and Janz 2010) [36]	Canada	To explore the cultural activities of the Métis population	Quantitative research using data from the 2006 Aboriginal Peoples Survey	N/A
**Grey Literature**				
(Métis Nation-Saskatchewan 2012) [37]	Saskatchewan	To describe a strategy to guide the work of the MN-S Health Department	Saskatchewan Métis Health Survey	1515 Métis residents of Saskatchewan
(Métis Nation-Saskatchewan 2023) [38]	Saskatchewan	To guide Métis cancer patients with their cancer journey by bringing hope, educating, sharing Métis cancer stories, and listing support services available to Métis citizens	Interviews	N/A
(Dorion 2010) [39]	Saskatchewan	To explore Cree and Métis Elders’ teachings about traditional child rearing and investigate how storytelling facilitates the transfer of this culturally based knowledge	Interviews	7 Cree and Métis Elders
(Ramsden et al., 2010) [40]	Saskatchewan	To build a framework that would conjointly engage the community and university partners in better understanding the social determinants of health within Métis communities	Saskatchewan Métis Health Survey and interviews	1,515 Métis residents of Saskatchewan
(Peterson 2023) [41]	Saskatchewan	To discuss how games help preserve Métis traditions	Interview	N/A
(Métis Nation-Saskatchewan 2012) [42]	Canada	To report on the health of Indigenous (Métis inclusive) peoples in Canada, including population health framework, determinants, indicators, and Métis Nation data collection efforts	Literature review	N/A
